# Re: Scragg–Emerging Evidence of Thresholds for Beneficial Effects from Vitamin D Supplementation

**DOI:** 10.3390/nu11061321

**Published:** 2019-06-13

**Authors:** Barbara J Boucher, William B. Grant

**Affiliations:** 1 The Blizard Institute, Barts & The London School of Medicine & Dentistry, Queen Mary University of London, London E1 2AT, UK; 2 Sunlight, Nutrition and Health Research Center, PO Box 641603, San Francisco, CA 94164-1603, USA; wbgrant@infionline.net

Scragg reports increasing evidence for differences in the thresholds for serum 25-hydroxyvitamin D [25(OH)D] concentration [vitamin D repletion] that need to be reached in populations observationally, or to be achieved by vitamin D supplementation in deficiency, before health benefits become apparent in different conditions [1]. Such variations are important as they add to the problems of developing adequate public health advice on desirable long-term vitamin D intakes and serum 25(OH)D concentrations at the population level. Additional evidence of such variations will assist in progressing the evolution of guidelines on optimal vitamin D intakes. Equally important will be the exclusion of confounding variations in threshold assessment due to differences in 25(OH)D assay methodology, since HMLC-TMS technology [2] normally gives higher results (often averaging about >5 nmol/L, but up to >33 nmol/L) than immunoassays [3]. Increasing compliance of laboratories providing 25(OH)D data with international quality control scheme requirements must be reducing assay unreliability. However, harmonization of older assay data with the newer “gold standard” methodology [4] will lead to the need to redefine the “cut-offs” classically used for defining deficiency and sufficiency. For example, standardised means of 49.7 nmol/ L (48.2–51.3) and 49.3 nmol/L (47.4–51.2) by immunoassay were 62.0 nmol/L (58.9–65.1) and 60.9 nmol/L (57.1–64.7) in women and men respectively, after standardization to LC–MS/MS methodology [3]. However, such standardization will also increase the possibility of including older reports in further assessments of thresholds for various health benefits of increasing vitamin D status. It may be useful, therefore, to mention some additional reports of thresholds for health risk reduction from the existing literature that could prove useful in further analyses of data relevant to this important topic. 

Reductions in abnormal insulin resistance have been reported after six months of supplementation with vitamin D at 4000 IU/day in deficient South-East Asian women living in New Zealand, but only in those women who achieved 25(OH)D values >80nmol/L [5], and, observationally, in obese black and white Americans at >65 nmol/L [6] and between 41.6 and 68.0 nmol/L in Hispanic black, Mexican American, and non-Hispanic white Americans [7]. Improved markers of bone health were found observationally with increasing serum 25(OH)D values >50 nmol/L in a study across 29 countries [8]. Literature review for features reducing fracture risks only found these at 25(OH)D values >50 nmol/L [9], while favourable responses to bisphosphonates in osteoporotic post-menopausal women required serum 25(OH)D concentrations >82 nmol/L [10]. Analyses of old and emerging data on thresholds for vitamin D efficacy using harmonized serum 25(OH)D concentrations, should, as Scragg points out, assist greatly in the development of optimal recommendations for long-term vitamin D_3_ intakes and serum 25(OH)D concentrations, which would allow rationalisation of public health decisions on recommended daily intakes, according to demonstrable biological effects of different levels of vitamin D status in humans. 
